# Whole Exome Sequencing of Biliary Tubulopapillary Neoplasms Reveals Common Mutations in Chromatin Remodeling Genes

**DOI:** 10.3390/cancers13112742

**Published:** 2021-06-01

**Authors:** Claudia Gross, Thomas Engleitner, Sebastian Lange, Julia Weber, Moritz Jesinghaus, Björn Konukiewitz, Alexander Muckenhuber, Katja Steiger, Nicole Pfarr, Benjamin Goeppert, Gisela Keller, Wilko Weichert, Nazmi Volkan Adsay, Günter Klöppel, Roland Rad, Irene Esposito, Anna Melissa Schlitter

**Affiliations:** 1 Institute of Pathology, School of Medicine, Technische Universität München, 81675 Munich, Germany; claudia_gross@gmx.de (C.G.); moritz.jesinghaus@tum.de (M.J.); bkonukiewitz1@gmx.de (B.K.); alexander.muckenhuber@tum.de (A.M.); katja.steiger@tum.de (K.S.); nicole.pfarr@tum.de (N.P.); gisela.keller@tum.de (G.K.); wilko.weichert@tum.de (W.W.); guenter.kloeppel@tum.de (G.K.); 2 Institute of Molecular Oncology and Functional Genomics, TUM School of Medicine, Technische Universität München, 81675 Munich, Germany; thomas.engleitner@tum.de (T.E.); sebastian.lange@tum.de (S.L.); julia.weber@tum.de (J.W.); roland.rad@tum.de (R.R.); 3 Center for Translational Cancer Research (TranslaTUM), TUM School of Medicine, Technische Universität München, 81675 Munich, Germany; 4 Department of Medicine II, Klinikum rechts der Isar, TUM School of Medicine, Technische Universität München, 81675 Munich, Germany; 5 German Cancer Consortium (DKTK), Partner Site Munich, 80336 Munich, Germany; 6 Institute of Pathology, University of Heidelberg, 69120 Heidelberg, Germany; benjamin.goeppert@med.uni-heidelberg.de; 7 Department of Pathology, School of Medicine, Koc University, Istanbul 34010, Turkey; adsay.volkan@gmail.com; 8 German Cancer Consortium (DKTK), German Cancer Research Center (DKFZ), 69120 Heidelberg, Germany; 9 Institute of Pathology, University Hospital of Duesseldorf, 40225 Duesseldorf, Germany; Irene.Esposito@med.uni-duesseldorf.de

**Keywords:** pancreas, bile duct, intraductal tubulopapillary neoplasms, whole exome sequencing, chromatin remodeling genes

## Abstract

**Simple Summary:**

Intraductal tubulopapillary neoplasms (ITPN) have recently been described as rare precursor lesions of pancreatic ductal adenocarcinoma and cholangiocarcinoma. Despite a high number of associated invasive adenocarcinomas at the time of diagnosis, patients with ITPN tend to have a much better clinical outcome than those with classical pancreato-biliary adenocarcinoma. Furthermore, rare molecular studies of ITPN show an unexpected lack of hotspot mutations in common driver genes of pancreato-biliary adenocarcinoma, including *KRAS.* This article reports the first large comprehensive and comparative molecular study of pancreato-biliary ITPN. In the absence of *KRAS* mutations, we found a high genetic heterogeneity with enrichment in core signaling pathways, including putative actionable genomic targets in one-third of the cases. Whereas, pancreatic ITPN demonstrates a highly distinct genetic profile, differing from classical pancreatic carcinogenesis, biliary ITPN and classical cholangiocarcinoma share common alterations in key genes of the chromatin remodeling pathway, and therefore, appear more closely related than pancreatic ITPN and classical pancreatic ductal adenocarcinoma PDAC.

**Abstract:**

The molecular carcinogenesis of intraductal tubulopapillary neoplasms (ITPN), recently described as rare neoplasms in the pancreato-biliary tract with a favorable prognosis despite a high incidence of associated pancreato-biliary adenocarcinoma, is still poorly understood. To identify driver genes, chromosomal gains and losses, mutational signatures, key signaling pathways, and potential therapeutic targets, the molecular profile of 11 biliary and 6 pancreatic ITPNs, associated with invasive adenocarcinoma in 14/17 cases, are studied by whole exome sequencing (WES). The WES of 17 ITPNs reveals common copy number variants (CNVs) broadly distributed across the genome, with recurrent chromosomal deletions primarily in 1p36 and 9p21 affecting the tumor suppressors *CHD5* and *CDKN2A*, respectively, and gains in 1q affecting the prominent oncogene *AKT3*. The identified somatic nucleotide variants (SNVs) involve few core signaling pathways despite high genetic heterogeneity with diverse mutational spectra: Chromatin remodeling, the cell cycle, and DNA damage/repair. An OncoKB search identifies putative actionable genomic targets in 35% of the cases (6/17), including recurrent missense mutations of the *FGFR2* gene in biliary ITPNs (2/11, 18%). Our results show that somatic SNV in classical cancer genes, typically associated with pancreato-biliary carcinogenesis, were absent (*KRAS*, *IDH1/2*, *GNAS*, and others) to rare (*TP53* and *SMAD4*, 6%, respectively) in ITPNs. Mutational signature pattern analysis reveals a predominance of an age-related pattern. Our findings highlight that biliary ITPN and classical cholangiocarcinoma display commonalities, in particular mutations in genes of the chromatin remodeling pathway, and appear, therefore, more closely related than pancreatic ITPN and classical pancreatic ductal adenocarcinoma.

## 1. Introduction

Pancreatic ductal adenocarcinomas (PDACs) and cholangiocarcinomas (CCAs) are highly aggressive neoplasms with a poor prognosis. In contrast, intraductal tubulopapillary neoplasms (ITPNs), recently described as rare precursor lesions of PDAC and CCA, tend to have a much better clinical outcome, despite a high number of associated invasive adenocarcinomas at the time of diagnosis [1,2]. Due to the rarity of the neoplasm, there are only a few studies on the clinical and genetic features of ITPNs [1,2,3,4,5,6]. In a previous study of 20 biliary ITPNs, we found a surprisingly low prevalence of alterations of driver genes commonly affecting PDAC and CCA, including *KRAS* (mutated in 6%), *GNAS*, *IDH1/2*, and *BRAF* (0%) in both the intraductal and invasive tumor components [1]. Based on the observed clinical, morphological, and molecular differences between biliary ITPNs and classical biliary adenocarcinomas, we hypothesized that ITPN tumor formation may occur through a distinct carcinogenesis pathway [1]. However, major pathways, key drivers, and potential druggable targets of biliary ITPN are still largely unknown. Recently, Basturk et al. published the first large molecular study of 22 pancreatic ITPNs, including whole genome sequencing (WGS) and whole exome sequencing WES of 5 cases, which revealed mutations in chromatin remodeling genes, genes of the phosphatidylinositol 3-kinase (PI3K) pathway, and *FGFR2* fusions [3].

To further elucidate the underlying genetic changes in biliary ITPNs, we studied 11 of these neoplasms and compared them with six pancreatic ITPNs. Using WES, we demonstrate a high genetic diversity with recurrent copy number variants (CNVs) (loss of chromosome 1p36 and others), and only a few recurrent somatic mutations, in ITPN (*TG* in 3/17; *SLIT2*, *FGFR2*, *HMCN1*, and others in 2/17). In addition, we identified the cell cycle, chromatin remodeling, and DNA damage/repair as key signaling pathways in both biliary and pancreatic ITPN, and highlighted targets for potential therapeutic options in 35% of the cases.

## 2. Materials and Methods

### 2.1. Study Cohort

The study was approved by the ethics committee of the TUM school of medicine, Technische Universität München, Munich, Germany (approval number 5593/12). Tumor samples from 17 patients were included in the study, and analyzed by WES.

All patients underwent surgical dissection for pancreato-biliary neoplasia; clinical details are given in Table A1. All cases were reevaluated by board-certified pathologists with a special interest in pancreato-biliary pathology (A.M.S., I.E., G.K., and V.N.A.), and histologically classified into ITPN of the bile duct (biliary ITPNs, *n* = 11) and ITPN of the pancreas (pancreatic ITPNs, *n* = 6), based on the recently proposed classification criteria [1,7]. Analyzed biliary ITPN cases from Europe, Asia, and the United States (labeled with ID 1–11) were previously characterized in a prior study. In detail, biliary ITPNs 1–11 corresponded to previously published case IDs 3, 4, 5, 7, 12, 13, 14, 15, 16, and 20, respectively [1]. In addition, six pancreatic ITPN cases from two German institutions, the Institute of Pathology, Technische Universität München (*n* = 3) and Institute of Pathology, University Heidelberg (*n* = 3), were included in the study.

### 2.2. Clinical Information and Statistical Analyses

Clinical, demographic, and macroscopic information was obtained from patient databases, and by reviewing medical charts and pathology reports. Follow up on the patients’ conditions was obtained from their clinical record, by directly contacting the patients and/or their physicians. Overall survival (OS) was defined as the time from resection until death, or until the last follow-up. Patients who suffered perioperative death (defined as death within the first 60 days after surgery) were excluded from further analyses. The OS of biliary ITPNs was compared to a matched cohort (*n* = 25) of previously published conventional intrahepatic (20/25) and extrahepatic/distal (5/25) cholangiocarcinoma without associated ITPN or IPNB [8,9]. The OS of pancreatic ITPNs was compared to a previously published cohort (*n* = 173) of resected PDACs [10]. Survival analysis was performed using the Kaplan–Meier method; differences were evaluated using the log-rank test. A two-sided *p*-value of <0.05 was considered significant.

### 2.3. Tumor Samples

Tumor DNA and matched normal DNA derived from non-neoplastic tissue distant from the tumor was isolated from formalin fixed paraffin embedded (FFPE) tissue, as described earlier [11]. Briefly, after de-paraffinization, tissue from areas with the highest tumor content (intraductal or combined intraductal and invasive) was chosen for manual microdissection, scraped off slides, and transferred into Qiagen ATL tissue lysis buffer (Qiagen GmbH, Hilden, Germany) supplemented with 10% proteinase K (20 mg/mL) solution.

Samples were incubated in a shaking heating block at 56 °C for 72 h, and fresh proteinase K solution (10% of total volume) was added every 24 h.

Subsequent downstream processing of samples was conducted using the Qiagen DNeasy Blood and Tissue Kit, according to the manufacturer’s instructions, with the following modification: Instead of the columns supplied in the kit, Qiagen MinElute spin columns were used, which allow elution in lower volumes (typically 30 µL), and therefore, more highly concentrated DNA samples. Sequencing libraries were prepared using the SureSelect Human All Exon V5 Kit, according to the manufacturer’s instructions. Libraries were sequenced on an Illumina HiSeq 2000/4000 sequencer system (lllumina, Inc., San Diego, California, USA), resulting in approximately 50 Mio. paired-end reads (2 × 100) per sample. The Genome Analysis Toolkit (GATK) Best Practice Guidelines [12] were followed for subsequent quality control and bioinformatic analyses. Detection of somatic nucleotide variants (SNVs) and CNVs in exonic regions was subsequently performed with Mutect2 [13] and CopywriteR [14], respectively.

### 2.4. SNV Calling

The WES data were processed as follows. Mutant and wildtype allele counts of individual somatic mutations at the DNA level were obtained from TCGA tumor BAM files. The sequencing coverage for SNV analysis was 60-fold on average. The annotation of mutations occurred via ensembl.org (accessed on 12 March 2019), version 78 [15]. Relevant mutations were selected manually by the individual threshold setting of the obtained sequencing reads (≥35), tumor alternate counts (≥3), and allelic frequencies (≥0.04) based on genomic coverage. Detailed information of each gene, such as the full name and function, was extracted from the National Center for Biotechnology Information (NCBI Entrez Gene Database) and PubMed (http://ncbi.nlm.nih.gov, accessed on 12 March 2019). Mutational signatures of each case were extracted on the basis of the identified SNV and their sequence context, as previously described ([16]; cancer.sanger.ac.uk/cosmic/signatures_v2http://ncbi.nlm.nih.gov, accessed on 12 March 2019). Resemblance to reported COSMIC signatures (cancer.sanger.ac.uk/cosmic/signatures_v2, accessed on 12 March 2019) was investigated using cosine similarity analysis. *OncoKB* (oncokb.org, accessed on 12 March 2019) supplied information on the known pathogenic effects of variants.

### 2.5. CNV Analysis

Chromosomal aberrations were identified via CopywriteR software [14]. For copy number information, off-target reads from targeted sequencing were used (R package version 2.12.0; downloaded via https://github.com/PeeperLab/CopywriteR, accessed on 1 February 2019). Log_2_ values within 20 kb bins in a range between ±0.2 and ±2.0 were defined as heterozygous amplifications or deletions, respectively. Log_2_ values above/below ±2.0 were defined as the homozygous status of amplifications or deletions, respectively. The plot CNV function allowed segmentation of the copy number data.

### 2.6. Fluorescence In Situ Hybridization (FISH) Analysis of Chromosome 1

The identified CNV of chromosome 1 (1p36 loss and 1q25 gain) were investigated by FISH analysis. FISH using Zytolight SPEC Dual Color 1p36/1q25 Probe (Z-2075-200, ZytoVision GmbH, Bremerhaven, Germany) was performed according to the manufacturer’s instructions [17].

## 3. Results

**Figure 1:**
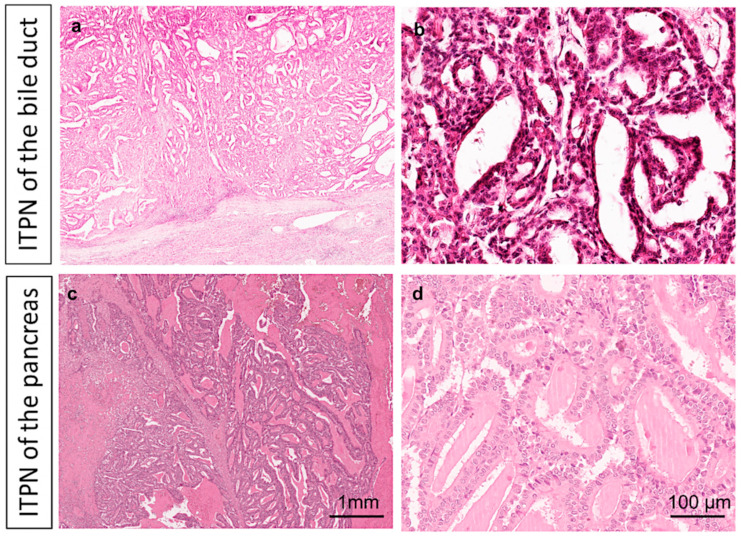
Histopathological hallmarks of intraductal tubulopapillary neoplasms (ITPNs). ITPNs of the bile duct (**a**,**b**) and pancreas (**c**,**d**) are characterized by intraductal tumor masses with predominant tubular growth in ITPN. Representative H&E staining at low (2×, **a**,**c**) and high (20×, **b**,**d**) magnification. Detailed clinical and histopathological information, as well as follow-up data of the study cohort, are given in Table A1.

To investigate the molecular profile of ITPN, we generated WES data from manually microdissected FFPE samples of 11 biliary and 6 pancreatic ITPNs (representative HE stainings are depicted in Figure 1).

**Figure 2:**
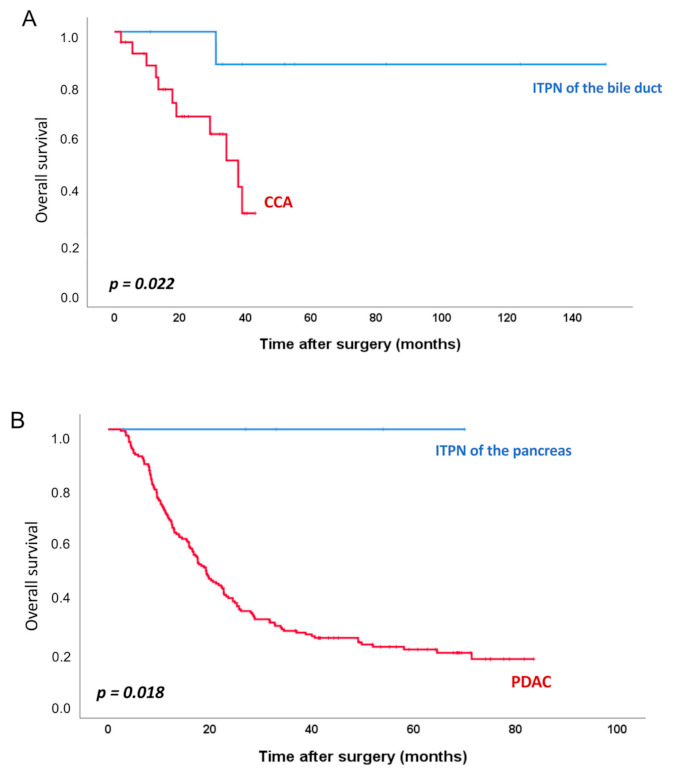
Survival analyses. Kaplan–Meier survival curves comparing overall survival of ITPN of the bile duct (*n* = 9) with cholangiocarcinoma (CCA) (*n* = 25) (**A**), and ITPN of the pancreas (*n* = 5) with pancreatic ductal adenocarcinoma (PDAC) (*n* = 173) (**B**).

Briefly, biliary ITPNs were predominantly located in the liver (9/11, 82%); extrahepatic cases were rare (2/11, 18%). Despite associated invasive adenocarcinoma in most cases (10/11 biliary ITPN; 4/6 pancreatic ITPN, and one case with features suspicious for invasion), the ITPNs had an excellent prognosis, with significantly better overall survival rates compared to classical pancreato-biliary adenocarcinoma (biliary ITPNs vs. classical CCAs: *p* = 0.022; pancreatic ITPNs versus classical PDACs: *p* = 0.018) (Figure 2).

### 3.1. Single Nucleotide Variants (SNVs)

**Figure 3:**
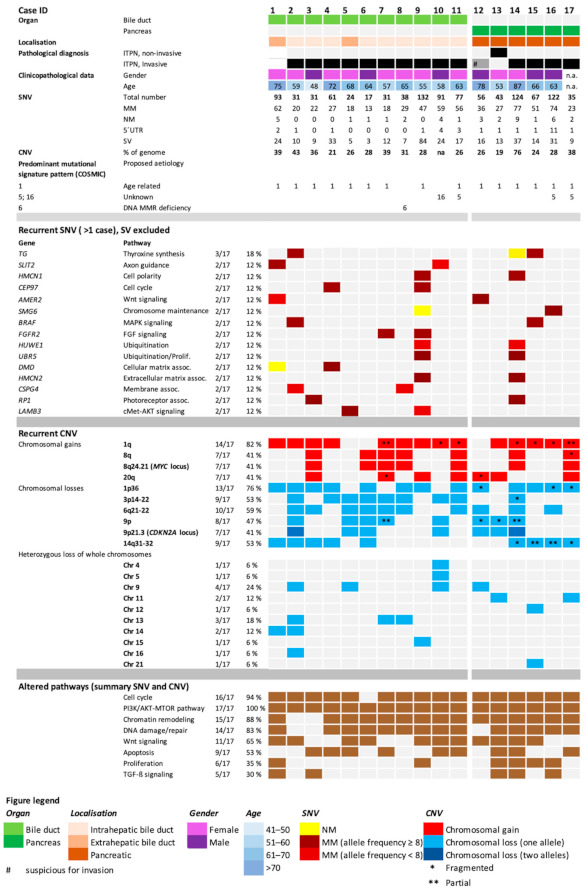
Oncoplot with clinicopathological features of individual patients and detailed molecular findings of whole exome sequencing (WES), including single nucleotide variants (SNVs), copy number variants (CNVs), mutational signature patterns, and altered key pathways. MM, missense mutation; NM, nonsense mutation; 5′UTR, mutation in the 5′untranslated region; SV, synonymous variant.

A detailed summary of the obtained results from CNV and SNV analyses is shown in Figure 3. The number of identified SNVs per sample (mean 63, ranging from 17 to 132), as well as the variation type (predominantly missense mutations), is shown in Figure 3. We identified a mean of 38 missense mutations in the cohort, ranging from 13 to 77.

### 3.2. Mutational Signature Pattern

**Figure 4:**
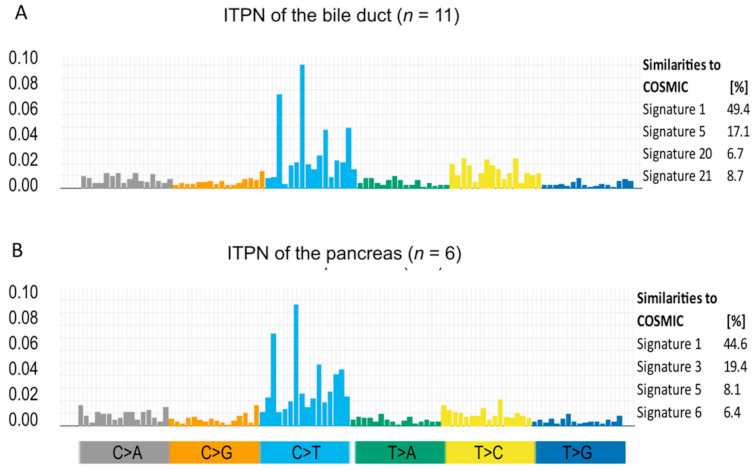
Mutational signature pattern analysis. Mutational signature patterns (left side) of ITPN of the bile duct (**A**) and of the pancreas (**B**), as well as their overlap with published COSMIC signatures (right side), show a resemblance to signature 1, the most common signature in all cancers, including pancreas and liver. Substitutions are displayed in the trinucleotide context and plotted on the *x*-axis, while fractions are shown on the *y*-axis. Each class of substitution is displayed by a different color.

In the next step, the obtained sequencing data were analyzed for mutational signatures. Briefly, analysis of the six different types of base substitutions in their immediate sequencing context was performed to generate individual mutational signatures, thus providing a deeper understanding of mutational processes [16]. Analysis of pancreato-biliary ITPN revealed a predominance of C>T mutations, with a prevalence at the NpCpG trinucleotides (Figure 4), closely resembling age-associated signature 1, the most common mutational signature across various human cancer types, including liver and pancreas cancer [16]. Interestingly, further analysis of individual cases confirmed the prevalence of signature 1 in all pancreatic ITPNs (6/6) and most biliary ITPNs (9/11) (Figure 4).

Signatures associated with DNA mismatch repair (MMR) deficiency (https://cancer.sanger.ac.uk/cosmic/signatures_v2, accessed on 10 May 2020), namely, signatures 6 and 21, were identified in one biliary ITPN (case # 8) harboring a STOP mutation in the tumor suppressor gene *BAP1*. Of note, none of the cases showed a predominance of signature 3, a signature associated with *BRCA1/2* mutations [16].

### 3.3. Recurrently Mutated Genes and Corresponding Pathways

Further analysis of identified SNVs showed a high genetic heterogeneity, with few recurrent mutations, in biliary and pancreatic ITPN. Recurrent SNV (defined as genes with mutations in more than one patient, excluding synonymous variants) are listed in Figure 3. The highest mutational frequency was observed in the TG gene (3/17, 18%). *FGFR2*, *AMER2*, *SLIT2*, *HMCN1*, *CEP17*, and others showed mutations in two tumors (2/17, 12%) (Figure 3). Genes that were affected by SNVs were mutated merely in one single tumor. No missense and nonsense mutations were found in *KRAS*, *CDKN2A*, *GNAS*, *IDH1/2*, and *EGFR*, genes associated with classical pancreato-biliary carcinogenesis (Table 1).

**Figure 5:**
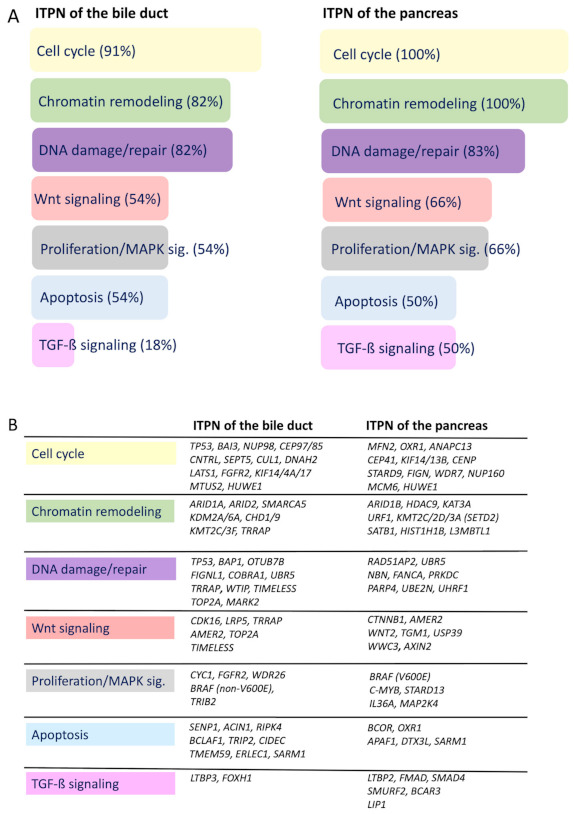
Identified key signaling pathways in ITPN carcinogenesis by SNV analysis (synonymous variants (SVs) excluded). Summary of commonly affected pathways by SNVs (% of all cases that showed SNVs in the respective pathways, SVs excluded) (**A**), and a detailed list of affected genes in the respective key signaling pathways (**B**).

Alterations in these classical drivers were restricted to CNV. Mutational inactivation of *TP53* was rare in biliary ITPN (1/11, 9%), and absent in pancreatic ITPN. Furthermore, no SNVs were detected in the cancer-predisposing genes *BRCA1/2*. Despite high genetic heterogeneity, deeper pathway analysis identified functional gene groups and defined core signaling pathways that were altered in the majority of tumors (Figure 5).

Pathway-wise, the highest frequencies of mutations were detected in chromatin remodeling (82–100%), the cell cycle (91–100%), and DNA damage/repair genes (82–83%) in biliary and pancreatic ITPN (Figure 5A). Most mutated genes involved in chromatin remodeling, along with *ARID1A/1B/2*, belonged to the SWI/SNF complex (*SMARCA5*) or regulated post-translational histone modification (*KDM2A*, *SETD2*, *HDAC9*). Furthermore, several cell cycle genes, such as the oncogene *FGFR2*, TP53-target genes *MFN2* and *BAI3*, tumor suppressors *BAP1* and *NUP98*, and DNA topoisomerase *TOP2A*, were affected. The DNA damage/repair pathway was affected by mutations in the aforementioned gene BAP1, the double strand break repair gene *PRKDC*, the cofactor of BRCA1 *COBRA1*, and the HAT recruiter *TRRAP*, to name just a few. In addition, 59% of all tumors showed mutations in genes affecting Wnt signaling (e.g., *AMER2, CTNNB1*, *CDK16*, *TIMELESS*, *WNT2*). Likewise, signaling pathways associated with apoptosis were affected in each group with mutations in the SUMO pathway-specific gene *SENP1*, chromatin condensation inducer gene *ACIN1*, and oxidation reductase gene *OXR1* (Figure 5B). In summary, chromatin remodeling, the cell cycle, and DNA damage/repair were identified as key oncogenic pathways in biliary and pancreatic ITPN.

### 3.4. Identified Potential Therapeutic Targets

The OncoKB database was used to search for actionable therapeutic targets identified by WES analysis. In total, putative actionable genomic targets were identified in 35% of the cases (6/17), including recurrent missense mutations of the *FGFR2* gene in biliary ITPN (2/11, 18%), as well as missense mutations in *BRAF* (V600E), *MTOR*, *NRAS,* and *KDM6A*. A detailed list of identified targets, including their level of evidence, is given in Table 2.

In addition, we identified common alterations of the Wnt signaling pathway in more than half of the cases (54% of biliary and 66% of pancreatic ITPNs), suggesting it is a signaling pathway with potential therapeutic opportunities [20]. One biliary ITPN carried a STOP mutation of *BAP1*, a gene that was previously suggested as a candidate for a predictive biomarker for immunotherapy of mesothelioma [21].

### 3.5. Genes Classically Associated with Pancreato-Biliary Carcinogenesis Are Rarely Altered in ITPN Carcinogenesis

Comparison of genetic profiles of biliary ITPN and pancreatic ITPN with those of CCA and PDAC [18,19] revealed that the typical genes associated with pancreato-biliary carcinogenesis were rarely affected (Table 2). Most striking was the complete absence of *KRAS* mutations in pancreatic ITPN (compared to reported mutation rates of >90% in PDAC). Likewise, no mutations of *TP53* were identified in this group, whereas biliary ITPN were enriched for mutations in chromatin remodeling genes associated with CCA (*ARID1A*, *KMT2C*, *BAP1*, and *ARID2*) (Table 1). The four key genes of PDAC carcinogenesis, namely, *KRAS*, *CDKN2A/p16*, *TP53*, and *SMAD4*, were mostly unaffected in pancreatic ITPN, except for a single loss of *SMAD4* and common loss of *CDKN2A*.

### 3.6. CNV Detects Recurrent Gains and Losses on Chromosome 1

**Figure 6:**
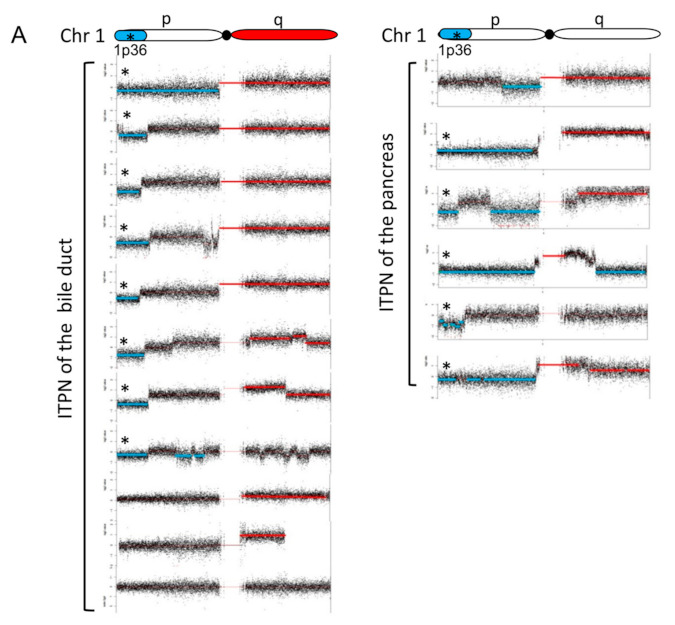

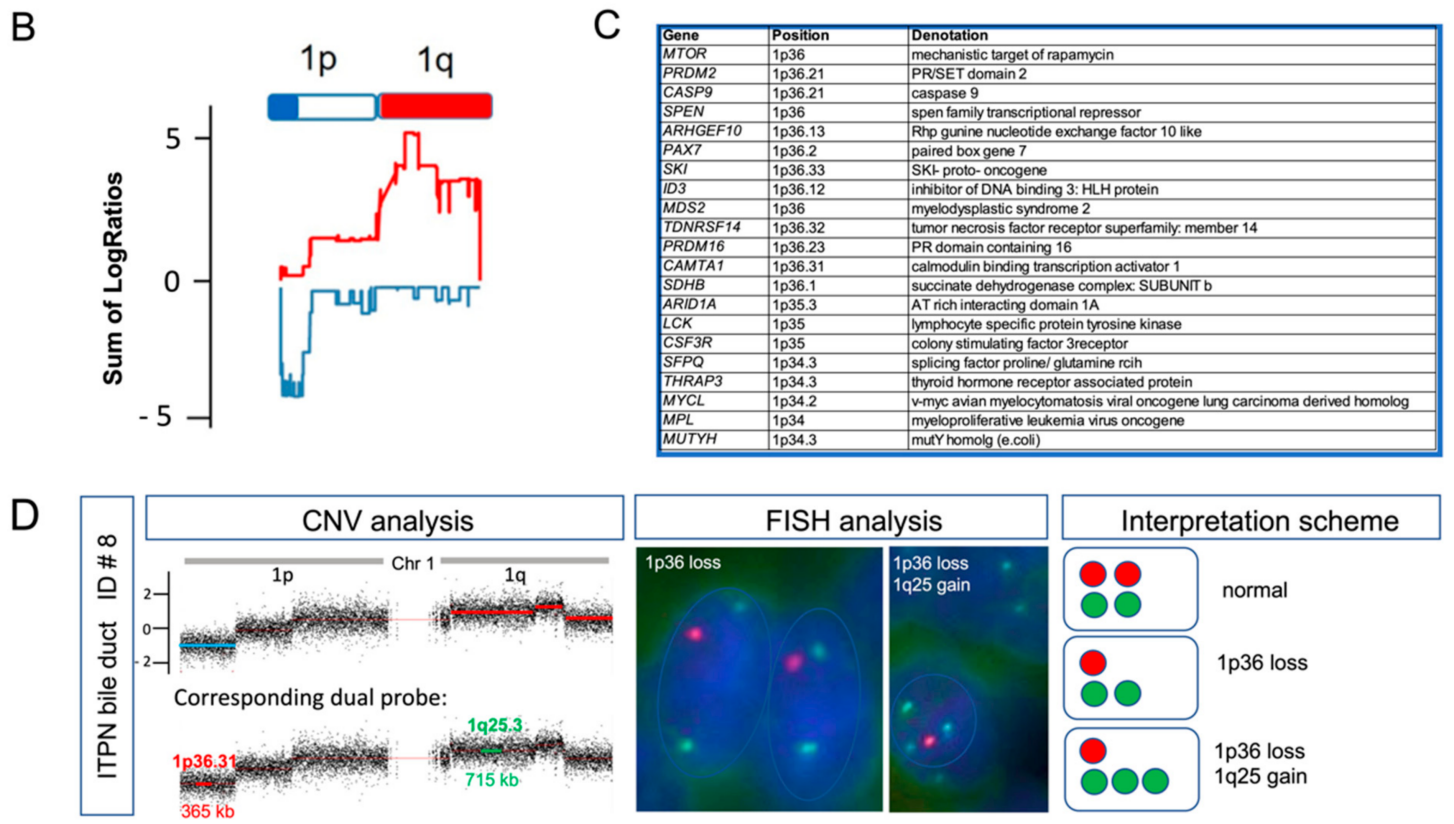
Recurrent chromosomal aberrations on chromosome 1 identified by CNV analysis. Individual plots of chromosome 1 of all cases (**A**). Deleted fragments of chromosome 1 are shown in blue (LogRatio ≤ −0.2), and amplifications are highlighted in red (LogRatio ≥ 0.2). Deletions were predominantly located at the telomeric 1p36 region (*), amplifications on 1q. Overlay-plot of chromosomal gains (red) and losses (blue) of all ITPNs (*n* = 17) (**B**). Gains and losses are displayed per chromosome (*x*-axis, chromosome 1–22) in summarized LogRatios (*y*-axis). Besides fragmental deletions and amplifications, single focal gains and losses are shown. This is an overview of cancer-associated genes located at the highly recurrent altered chromosomal region 1p34-36 (extracted from COSMIC cancer gene consensus, https://cancer.sanger.ac.uk/census, accessed on 12 March 2019) (**C**). Fluorescence in situ hybridization (FISH) analysis of chromosome 1 (biliary ITPN, ID #8) (**D**). The illustration on the left side shows the individual plot and the dual FISH probe targeting regions 1p36 and 1q25. Fluorescence microscopy confirmed the heterozygous loss of 1p36. The interpretation scheme is depicted on the right side.

CNVs were detected in all cases, ranging from an estimated 21–76% of the genome. On average, ITPN showed CNVs in 34.5% (pancreatic) and 31.5% (biliary) of the genome. Moreover, distinct patterns of chromosomal aberrations were identified in pancreatic and biliary ITPN. Large recurrent fragment damage, ranging from 16 to 45 MB, was observed in biliary ITPN on chromosomes 1, 3, 6, 9, and 14, whereas smaller recurrent fragmental damage (between 2 and 23 MB) was identified in pancreatic ITPN on chromosomes 1, 11, 12, 14, and 20. Interestingly, recurrent chromosomal losses and gains within specific regions were identified on chromosome 1, with chromosomal gains on the long arm 1q (14/17, 82%) and chromosomal losses on region 1p36 (13/17, 76%), a region that contains numerous cancer-associated genes (Figure 6; available at COSMIC: https://cancer.sanger.ac.uk/census, accessed on 12 March 2019) (for details see Figure 3 and Figure 6). To further validate the obtained CNV changes on chromosome 1, an additional FISH analysis was performed on individual cases using a dual 1p36/1q25 probe, which confirmed the observed heterozygous loss of 1p36 and low-level amplification of 1q in a majority of tumor cells (Figure 6).

### 3.7. Genetic Losses

In addition to recurrent deletions of 1p36, further genetic losses were frequently detected on chromosomes 3, 6, 9, and 14 (Figure 3). On chromosome 3, 53% of the analyzed tumors showed deletion of region 3p12-22, with a fragmental loss of 47 MB. In three cases of analyzed biliary ITPNs, the complete 3p arm was lost heterozygously (88 MB), a region that contains several common tumor suppressor genes (e.g., *BAP1*).

The long arm of chromosome 6 was deleted in 73% of biliary and in 33% of pancreatic ITPNs. The chromatin remodelers *ARID1B* and *HDAC2*, as well as genes involved in the cell cycle (*BCLAF1*, *BAI3*) and HIPPO pathway (*LATS1*), are located in that altered region.

In addition, chromosome 9 revealed recurrent CNVs in pancreato-biliary ITPN. A total of 53% of all ITPNs showed chromosomal aberrations on chromosome 9; in 45% of biliary ITPNs (5/11), the long arm of chromosome 9 (9q) was affected, and in some pancreato-biliary ITPN cases (4/17, 24%), the whole chromosome was deleted. *TGFBR1* represents one prominent gene affected by 9q depletion. Seven of seventeen cases (41%, thereof 4/11 biliary ITPN and 3/6 pancreatic ITPN) showed a loss of 9p21. In all, 5/17 cases (33%) showed a heterogeneous loss of 9p21, and 2/17 (10%) showed a biallelic inactivation of that region, containing the prominent tumor suppressor gene *CDKN2A*. A full 47% of analyzed pancreato-biliary ITPNs showed a genetic loss on chromosome 12 without any recurrent pattern. In one pancreatic ITPN, heterogeneous deletion of the whole chromosome was observed, encompassing a region that contains numerous cancer-specific genes, including the classical cancer genes *KRAS*, *MDM2*, *ARID2*, *KDM5A*, *TIMELESS*, and *WNT*. Chromosome 14 revealed deletions in 53% of all analyzed ITPN samples, and 24% of these samples showed a complete heterogeneous chromosomal arm loss (position 20 to 92 MB). In the remaining 29%, only a fragmental loss of 14q was found. Several known cancer-associated genes are located on 14q, including *CHD8*, *FOXA1*, *HIF1A*, and *DICER1*, encoding proteins involved in DNA damage/response, hypoxia response, and miRNA processing, respectively.

Small focal deletions, ranging in size between 10 and 50 kb, were found on different chromosomes, but mostly in non-coding regions, except one homozygous loss of the *UGT2B17* gene (UDP glucuronosyltransferase family 2-member B17, an enzyme-specific for the steroid metabolism) on chromosome 4 in one biliary ITPN. In contrast, loss of large regions was more common: Heterozygous loss of whole chromosomes was found in 6/11 biliary and 4/6 pancreatic ITPNs (Figure 3).

### 3.8. Genetic Gains

Recurrent genetic gains in pancreato-biliary ITPN were located on chromosomes 1q, 8q, and 20q (Figure 3). The most frequent recurrent aberration was found in region 1q (Figure 3). Amplifications of that chromosomal fragment were identified in 82% of all ITPN samples (9/11 biliary and 5/6 pancreatic ITPNs), and 50% of pancreatic ITPNs showed a high level of amplifications, defined as a LogRatio of >2. In biliary ITPN, high level amplification was observed in a minority of cases. In pancreato-biliary ITPN, the amplified region on chromosome 1q implicated the chromosomal band 1q21, a region that contains specific genes involved in histone/chromatin modification, such as *H3F3A*, *KDM5B*, *KMT2H*, and TADA1. In addition, the region harbors the prominent cancer gene and negative TP53-regulator *MDM4*, and the oncogene *AKT3*.

A recurrent gain was found on chromosome 8q. A total of 41% of pancreato-biliary ITPNs showed a heterogeneous amplification of that region, which contains different cancer-specific genes (e.g., oxidation resistance 1 *OXR1*, and the prominent proto-oncogene *MYC*).

Frequent CNV was observed on chromosome 20. In 7 of 17 cases (41%), 20q, a chromosomal region associated with neuroendocrine pancreatic tumors [22], and commonly amplified in gastric adenocarcinomas, was amplified [23]. Moreover, amplification of chromosome 20 has been associated with the immortalization of cell lines [24].

## 4. Discussion

Comprehensive molecular studies in conventional PDAC and CCA have provided insights into their carcinogenesis and identified common driver genes, subtypes with special prognostic features, and therapeutic targets [18,19,25]. In contrast, the molecular carcinogenesis of biliary ITPN, recently described as a rare precursor of CCA, is still poorly understood. Previous molecular studies on biliary ITPN have been restricted to selected genes and have failed, except for the identification of recurrent alterations of *CDKN2A/p16*, to detect significantly mutated genes or potential druggable targets [1,4]. In a recent study of 22 pancreatic ITPNs by Basturk et al. based on targeted NGS and/or WES and WGS in a handful of selected cases, alterations in genes related to chromatin remodeling, PI3K signaling, and *FGFR2* fusions were found [3], while mutations that are common in IPMN of the pancreas were lacking.

Here we report the first large comprehensive molecular study based on WES analyses of 11 biliary and 6 pancreatic ITPNs. Excellent overall survival, significantly better than matched patient cohorts with conventional pancreato-biliary adenocarcinomas, was observed in ITPN patients despite an association with invasive adenocarcinoma in most cases at diagnosis. Using WES analyses, we identified an average mutation burden of 56 (range 17–132) in biliary and 75 (range 43–124) in pancreatic ITPN, with a predominance of missense mutations, which is in line with published WES analyses of solid tumors, including PDAC, intrahepatic CCA, and small cell lung cancer, with reported mutation rates between 40 and 101 [25,26,27]. Our data clearly revealed and confirmed that biliary and pancreatic ITPNs generally lack *KRAS* and *GNAS* mutations, a remarkable finding given the crucial role of *KRAS* in the carcinogenesis of PDAC (mutated in >90% of cases), its precursor lesions (e.g., IPMN) and CCA (mutated in 17% of cases), and recurrent *GNAS* mutations in pancreatic IPMNs. Interestingly, a pathogenic mutation of the oncogene and RAS-family member *NRAS* (G12D) was detected in one pancreatic ITPN (1/6). In the absence of *KRAS* and *GNAS* mutations, we found a high genetic heterogeneity, with only a few low recurrent mutations in selected genes (*TG*, *FGFR2*, *SLIT2*, *AMER2*, *HMCN1,* and others), and missense mutations of *FGFR2*, *AMER2*, *SLIT2*, *CEP97*, *LAMB3*, *CSPG4*, and *DMD* in 18% of biliary ITPNs. The finding of recurrent missense mutations in the *FGFR2* gene is of particular clinical interest given the therapeutic implications for activating mutations in this gene for treatment of solid tumors, including CCA, with FGFR inhibitors [28] (classified as level 4 (compelling biological evidence) according to OncoKB search), as well as the reported finding of recurrent *FGFR2*-fusions in 22% of pancreatic ITPNs [3]. Of note, no mutations in the *FGFR2* gene were identified in our pancreatic ITPN cases. Lately, druggable fusions of this gene have been identified in small subgroups of patients with classical PDAC (e.g., *NTRK* or *ROS1* fusions) and among patients with *KRAS*-wildtype PDAC [29]. Although we identified several novel and known genetic alterations in our study, our WES approach did not address this interesting topic. Further studies using specific fusion detection strategies are, therefore, needed to investigate the relevance of these actionable fusions in biliary ITPN.

In total, putative actionable genomic targets (*FGFR2*, *BRAF-V600E*, *MTOR*, *NRAS*, and *KDM6A*) were identified in 35% of the cases by OncoKB search, an important finding given the high number of fluent transitions from high-grade IEN to adenocarcinoma at the time of diagnosis (14/17 cases). In addition, more than half of the cases harbored mutations in genes associated with Wnt signaling, a pathway recently recognized for its potential for therapeutic intervention [20]. Moreover, a STOP mutation of *BAP1*, previously suggested as a candidate predictive biomarker for immunotherapy of mesothelioma [21], and an emerging therapeutic option, was detected in one biliary ITPN. Recently, a newly generated and thoroughly characterized cell line from pancreatic ITPNs harboring many somatic mutations (including in genes involved in DNA repair and Wnt signaling) and structural rearrangements were reported, that could be used as in vitro model to further investigate actionable targets of ITPNs [30].

In general, genes classically associated with pancreato-biliary carcinogenesis, except the loss of *CDKN2A/p16* that we detected in more than 40% of all ITPNs, were rarely mutated in ITPN. Out of the 20 most commonly mutated genes in CCA reported in the literature [18], only six genes were altered in biliary ITPN: *TP53* at a very low frequency, *BRAF* (non-V600E), and chromatin remodeling genes (*ARID1A*, *ARID2*, *KTM2C*, and *BAP1*). In contrast, *SMAD4* and *CDKN2/p16* were the only genes out of the 20 most commonly mutated/altered genes in PDAC reported in the literature [19] identified in pancreatic ITPNs. These findings emphasize the notion that biliary and pancreatic ITPNs show a distinct *KRAS*- and predominantly *TP53*-independent genetic profile. However, our findings indicate that both biliary ITPN and classical cholangiocarcinoma (CCA) share common alterations in key genes of the chromatin remodeling pathway. In contrast, pancreatic ITPN demonstrates a highly distinct genetic profile, with only a few commonalities differing from classical pancreatic carcinogenesis.

Mutational signature analysis of the obtained sequencing data was used to obtain an understanding of mutational processes that play a role in the tumorigenesis of ITPN [16]. The majority of cases showed an age-related signature (signature 1), which is in line with the advanced age of the patients (median age of diagnosis was 63 years for biliary cases (range 48–75) and 66 for pancreatic ITPNs (range 53–87)). This signature, which is the most common mutational signature across various human cancer types, including those of the liver and pancreas, seems to result from endogenous mutational processes initiated by spontaneous deamination of 5-methylcytosine [16]. In addition, one biliary ITPN with a pathogenic STOP mutation in the *BAP1* gene, which plays a role in the BRCA1 growth control pathway, showed a signature pattern associated with DNA MMR deficiency.

Deeper pathway analysis of biliary and pancreatic ITPNs revealed the cell cycle (91–100%), chromatin remodeling (82–100%), and DNA damage/repair (82–83%) as core signaling pathways in both biliary and pancreatic ITPNs. These results are in line with the data of Basturk’s study, which showed common alterations in genes of the chromatin remodeling pathway in pancreatic ITPNs [3]. In addition, we identified mutations in genes affecting Wnt signaling (e.g., *CTNNB1*, *AMER2*) in more than 50% of biliary (54%) and pancreatic ITPNs (66%).

Further analysis addressed chromosomal changes in ITPNs. CNVs are critical genetic events that contribute to carcinogenesis, and frequently occur in all cancer types. However, the landscape of chromosomal variations has not been addressed in biliary ITPN to date. In our study, exome wide copy number analysis was performed to detect chromosomal gains and losses. In total, a moderate level of CNVs was detected in the majority of cases. In detail, calculated CNV levels ranged from moderate (21% of the genome) to a high fraction of the genome (76%). Deeper analysis revealed common CNVs broadly distributed among the genome, with recurrent chromosomal deletions affected in more than one-third of the cases, respectively, of 1p36, 3p14-22, 6q21-22, 9p21, and 14q21-22, as well as gains of 1q, 8q, and 20q. Loss of 9p21, which contains the *CDKN2A/p16* locus, a gene commonly affected in pancreato-biliary carcinogenesis and considered a key driver of PDAC, was detected in a high proportion of biliary ITPNs (36%) and half of pancreatic cases (50%). Our data are in line with previously published data that identified recurrent loss of heterozygosity in chromosome 1p and recurrent gains in chromosomes 1q and 8 in pancreatic ITPN [3]. Likewise, the observed spectrum of chromosomal copy number alterations showed a high concordance with reported patterns in intrahepatic cholangiocarcinoma (deletions in 1p, 3p, and 14q and gains in 1q, 7p, 7q, and 8q [31]). The majority of ITPNs showed the loss of 1p36 (13/17 cases) and gain of 1q (14/17 cases). Deletions of 1p were first reported in neuroblastoma in 1977. Since that time, numerous reports have shown that the 1p36 locus is commonly deleted in a variety of human cancers, including neoplasms of the colon, breast, thymus, and bone marrow, to name a few, suggesting a tumor suppressor function [32]. The locus contains numerous cancer-associated genes (*CASP9*, *SDHB*, and others), including chromodomain helicase DNA binding domain 5 (the *CHD5* gene), which was recently identified as a tumor suppressor in vivo [33]. Gain of 1q is a frequently observed genetic aberration in various hematologic malignancies and solid tumors, including many pediatric tumors (e.g., Wilms tumors), and is commonly associated with a poor prognosis [34].

Overall, our results showed high consistency with the abovementioned pancreatic ITPN study by Basturk et al. [3]. Our study confirmed their findings of common gene amplifications and deletions, in particular recurrent loss of heterozygosity in chromosome 1p, recurrent gains in chromosomes 1q and 8, the common loss of *CDKN2A*, and common alterations in chromatin remodeling genes and genes of the Wnt signaling pathway in an independent pancreatic ITPN cohort. Moreover, our study highlighted for the first time that biliary and pancreatic ITPN share these common molecular alterations. The main differences are a lack of *PIK3C* mutations found in our study, in both pancreatic and biliary cases, in contrast to reported high *PIK3C* mutation rates in previous studies by Basturk et al. [3] and Yamaguchi et al. [6].

## 5. Conclusions

Biliary and pancreatic ITPN share an excellent prognosis and a *KRAS*-independent genetic profile, characterized by genetic heterogeneity with enrichment in core signaling pathways affecting the cell cycle, chromatin remodeling, and others, and potential druggable targets in 35% of the cases. However, our findings highlighted that biliary ITPN and classical cholangiocarcinoma display commonalities, in particular mutations in genes of the chromatin remodeling pathway, and appear, therefore, more closely related than pancreatic ITPN and classical PDAC.

## Figures and Tables

**Table 1:** Comparison of identified alterations in ITPNs, with reported mutation rates of classical pancreato-biliary adenocarcinomas. List of reported most common mutated genes in cholangiocarcinoma (CCA) [18] (left side) and pancreatic ductal adenocarcinoma (PDAC) [19] (right side).

Bile Duct			Pancreas		
Gene	Reported Mutation Rates in CCA [18]	Observed Frequency (SNV) in ITPN of the Bile Duct	Gene	Reported mutation Rates in PDAC [19]	Observed Frequency (SNV) in ITPN of the Pancreas
*TP53*	26%	9%	*KRAS*	92%	0%
*KRAS*	17%	0%	*TP53*	45%	0%
*SMAD4*	8%	0%	*SMAD4*	17%	17%
*NF1*	6%	0%	*FLG*	10%	0%
*ARID1A*	6%	9%	*ATXN1*	7%	0%
*PBRM1*	6%	0%	*COL14A1*	6%	0%
*KMT2D*	6%	0%	*ITGAE*	6%	0%
*ATR*	6%	0%	*GLI3*	6%	0%
*PIK3CA*	5%	0%	*GNAS*	6%	0%
*ERBB3*	5%	0%	*SPTA1*	6%	0%
*KMT2C*	5%	9%	*ARID1A*	5%	0%
*PIK3C2G*	4%	0%	*CDKN2A*	5%	0% *
*APC*	4%	0%	*RP1L1*	5%	0%
*BAP1*	4%	9%	*BCLAF1*	5%	0%
*POLQ*	4%	0%	*AXIN1*	5%	0%
*ARID2*	4%	9%	*NIN*	5%	0%
*IDH1*	3%	0%	*RNF43*	4%	0%
*TET1*	3%	0%	*RBM10*	4%	0%
*CTNNB1*	3%	0%	*IRF2*	4%	0%
*BRAF*	3%	9%	*HDAC2*	3%	0%

* (CNV loss 50%).

**Table 2:** Summary of identified known and potential therapeutic targets. The OncoKB database was used to search for actionable therapeutic targets.

Identified Actionable Genes				Case ID	OncoKB Search		
Pathway	Gene	Alteration	Entity		Drugs	Level of Evidence	Level-assoc. Cancer Types
MAPK signaling	*BRAF*	p.Val600Glu	ITPN pancreas (1/6)	# 2	Vemurafenib, Dabrafenib, Trametinib	1	Melanoma
					Encorafenib + Cetuximab	1	Colorectal cancer
	*NRAS*	p.Gly12Asp	ITPN pancreas (1/6)	# 12	Binimetinib, Combimetinib, Iodine I 131-6-Beta-Iodomethyl-19-Norcholesterol + Selumetinib	3A	Melanoma, Histiocytosis, Thyroid cancer
					Panitumumab, Cetuximab	R1	Colorectal cancer
MTOR signaling	*MTOR*	p.Ser1863Leu*	ITPN pancreas (1/6)	# 14	Everolimus, Temsirolimus	4	All solid tumors
FGF signaling	*FGFR2*	p.Ile355Arg* p.Ile643Arg*	ITPN bile duct (2/11)	# 7,# 9	Erdafitinib, Debio 1347, AZD4547, BGJ398	4	All solid tumors
Chromatin remodeling	*KDM6A*	p.Ser440Leu*	ITPN bile duct (1/11)	# 8	Tazemetostat	4	Bladder cancer

Alterations marked with an asterisk (*) are not listed in the respective database. OncoKB levels of evidence are defined as level 1—FDA-approved; level 2—standard care; level 3A—clinical evidence; level 4—biological evidence; R1—resistance.

## Data Availability

Data are available upon request.

## Appendix A

**Table A1:** Detailed clinical, pathological, and follow-up data of ITPN patients.

Case ID #	Age at Diagnosis	Gender	Diagnosis	Invasive Tumor	Tumor Size (cm)	pT	pN	Survival (Months)	Status
1	75	w	ITPN of the bile duct	no	7.5	n.a.	n.a	31	death
2	59	w	ITPN of the bile duct	yes	6.0	pT1	pN0 (0/20)	33	alive at last follow-up
3	48	m	ITPN of the bile duct	yes (minimal invasive)	15.0	n.a.	n.a	83	alive at last follow-up
4	72	w	ITPN of the bile duct	yes	6.7	n.a.	pN0 (0/4)	52	alive at last follow-up
5	68	w	ITPN of the bile duct	yes	5.5	n.a.	pN0 (0/1)	124	alive at last follow-up
6	64	m	ITPN of the bile duct	yes	9.0	n.a.	n.a	1	perioperative death
7	57	w	ITPN of the bile duct	yes	7.0	pT2b	pN1 (1/24)	39	alive at last follow-up
8	65	w	ITPN of the bile duct	yes	5.0	n.a.	pN0 (0/4)	55	alive at last follow-up
9	55	w	ITPN of the bile duct	yes	7.0	n.a.	pN0 (0/5)	1	perioperative death
10	58	m	ITPN of the bile duct	yes	5.0	n.a.	n.a	11	alive at last follow-up
11	78	w	ITPN of the bile duct	yes	n.a.	n.a.	n.a	150	alive at last follow-up
12	53	m	ITPN of the pancreas	suspicious for invasion	3.5	pTis	pN0 (0/21)	27	alive at last follow-up
13	87	w	ITPN of the pancreas	no	15.0	pTis	pN0	33	alive at last follow-up
14	66	w	ITPN of the pancreas	yes	10.5	pT2	pN0 (0/24)	3	alive at last follow-up
15	63	m	ITPN of the pancreas	yes	6.0	pT3	pN1 (1/17)	70	alive at last follow-up
16	76	m	ITPN of the pancreas	yes	4.0	pT3	pN1	54	alive at last follow-up
17	63	w	ITPN of the pancreas	yes	1.8	pT1c	pN1 (1/17)	0	perioperative death
